# Developing an online, searchable database to systematically map and organise current literature on retention research (ORRCA2)

**DOI:** 10.1177/17407745211053803

**Published:** 2021-10-24

**Authors:** Anna Kearney, Polly-Anna Ashford, Laura Butlin, Thomas Conway, William J Cragg, Declan Devane, Heidi Gardner, Daisy M Gaunt, Katie Gillies, Nicola L Harman, Andrew Hunter, Athene J Lane, Catherine McWilliams, Louise Murphy, Carrie O’Nions, Edward N Stanhope, Akke Vellinga, Paula R Williamson, Carrol Gamble

**Affiliations:** 1 Health Data Science, University of Liverpool, Liverpool, UK; 2 Norwich Clinical Trials Unit, University of East Anglia, Norwich, UK; 3 Centre for Trials Research, Cardiff University, Cardiff, UK; 4 HRB-Trials Methodology Research Network and Evidence Synthesis Ireland; 5 Clinical Trials Research Unit, Leeds Institute of Clinical Trials Research, University of Leeds, Leeds, UK; 6 HRB-Trials Methodology Research Network, School of Nursing and Midwifery, NUI Galway, Galway, Ireland; 7 Health Services Research Unit, University of Aberdeen, Aberdeen, UK; 8 Population Health Sciences, Bristol Medical School, University of Bristol, Bristol, UK; 9 School of Nursing and Midwifery, National University of Ireland Galway, Galway, Ireland; 10 National University of Ireland Galway, Galway, Ireland; 11 University College Birmingham, Birmingham, UK; 12 Staffordshire University, Stoke-on-Trent, UK; 13 School of Medicine, National University of Ireland Galway, Galway, Ireland; 14 Liverpool Clinical Trials Centre, University of Liverpool, Liverpool, UK

**Keywords:** Participant retention, clinical trials, trials methodology, attrition, literature review

## Abstract

**Background:**

Addressing recruitment and retention challenges in trials is a key priority for methods research, but navigating the literature is difficult and time-consuming. In 2016, ORRCA (www.orrca.org.uk) launched a free, searchable database of recruitment research that has been widely accessed and used to support the update of systematic reviews and the selection of recruitment strategies for clinical trials. ORRCA2 aims to create a similar database to map the growing volume and importance of retention research.

**Methods:**

Searches of Medline (Ovid), CINAHL, PsycINFO, Scopus, Web of Science Core Collection and the Cochrane Library, restricted to English language and publications up to the end of 2017. Hand searches of key systematic reviews were undertaken and randomised evaluations of recruitment interventions within the ORRCA database on 1 October 2020 were also reviewed for any secondary retention outcomes. Records were screened by title and abstract before obtaining the full text of potentially relevant articles. Studies reporting or evaluating strategies, methods and study designs to improve retention within healthcare research were eligible. Case reports describing retention challenges or successes and studies evaluating participant reported reasons for withdrawal or losses were also included. Studies assessing adherence to treatments, attendance at appointments outside of research and statistical analysis methods for missing data were excluded. Eligible articles were categorised into one of the following evidence types: randomised evaluations, non-randomised evaluations, application of retention strategies without evaluation and observations of factors affecting retention. Articles were also mapped against a retention domain framework. Additional data were extracted on research outcomes, methods and host study context.

**Results:**

Of the 72,904 abstracts screened, 4,364 full texts were obtained, and 1,167 articles were eligible. Of these, 165 (14%) were randomised evaluations, 99 (8%) non-randomised evaluations, 319 (27%) strategies without evaluation and 584 (50%) observations of factors affecting retention. Eighty-four percent (n = 979) of studies assessed the numbers of participants retained, 27% (n = 317) assessed demographic differences between retained and lost participants, while only 4% (n = 44) assessed the cost of retention strategies. The most frequently reported domains within the 165 studies categorised as ‘randomised evaluations of retention strategies’ were participant monetary incentives (32%), participant reminders and prompts (30%), questionnaire design (30%) and data collection location and method (26%).

**Conclusion:**

ORRCA2 builds on the success of ORRCA extending the database to organise the growing volume of retention research. Less than 15% of articles were randomised evaluations of retention strategies. Mapping of the literature highlights several areas for future research such as the role of research sites, clinical staff and study design in enhancing retention. Future studies should also include cost–benefit analysis of retention strategies.

## Introduction

The recruitment and retention of participants are key challenges for conducting clinical trials, leading to a significant waste of research resources and funding.^1^ Participant retention is defined as all randomised participants continuing in the study and providing outcome data until the end of the study. While meeting recruitment targets is often a key concern for trials, funders and governing bodies,^2^ these efforts are wasted if participants are not subsequently retained and do not contribute outcome data for the analysis. Failing to retain participants may be more damaging than failing to recruit them in the first place due to the potential for biased estimates of treatment effect if losses are unbalanced between arms or are associated with the outcome being measured.^3^

Several studies have tried to estimate the extent of retention problems through analysis of levels of missing data reported in publications. Approximately 81%–95%^4–6^ of trials report some level of participant attrition and missing data. Across different cohorts of publications, median levels of missing data vary between 6% and 12%^4–9^ of all randomised participants within a trial. However, levels vary substantially with some trials reporting upwards of 55%.^5,7–9^ Reporting of retention is also acknowledged to be suboptimal, suggesting that the problem may be much greater.^10–13^

While statistical tools exist to handle missing data, these rely on assumptions about the nature and pattern of the missingness.^14^ Consequently, there is often debate about the most appropriate method^5,6^ and the subsequent validity of the estimated treatment effect.^4^ Prevention is better than cure. As recommended by the US Food and Drug Administration (FDA) and European Medicines Agency (EMA), the need for statistical techniques should be minimised by embedding strategies within the trial design to improve participant retention and maximise data collection.^14^ However, there is a paucity of evidence to support the selection and use of such strategies. Systematic reviews of randomised and non-randomised evaluations of retention interventions identified several studies aimed at improving questionnaire return but few addressing other causes of attrition^15,16^ such as failure of participants to attend study visits where data are collected. Similarly, many retention strategies regularly used by UK Clinical Research Collaboration (UKCRC) Registered Clinical Trial Unit Network had no known evidence to show their effectiveness.^17^

Evidence-based trial design and conduct and the trials methodology research underpinning it are essential for minimising research waste. In 2014, addressing recruitment and retention challenges was identified as a top methodological research priority,^18^ adding further momentum to the exponential growth of published literature in these areas.^8^ However, identifying relevant literature through search engines can be difficult and time-consuming, especially for those seeking to identify solutions for a specific participant demographic or trial design.

ORRCA (Online Resource for Research in Clinical triAls, www.orrca.org.uk), a free online and searchable database of recruitment research, was created in 2016^19^ to help trialists and methodologists quickly identify relevant literature. The database is updated regularly and has been accessed by users from across the world and has supported trial development and the update and delivery of methodological systematic reviews.^20–23^ However, no such resource exists for quickly and easily accessing retention research.

In this article, we aim to describe the methods used to develop a database of retention research and present a mapping of the literature to identify effective retention strategies and areas for future research.

## Methods

### Search strategies and identification of the literature

The search strategy aimed to identify peer-reviewed research relevant to retention within randomised controlled trials (RCTs). The search strategy used in the Cochrane Methodology Review of retention interventions^15^ was adapted incorporating elements from a similar review^16,24^ and additional terms for specific retention strategies beyond questionnaire return. The search strategy comprised two components, terms relating to retention and retention strategies and RCT terms.

The strategy was tested against a selection of key papers provided by the advisory team before being adapted for use with Medline (Ovid) (1946–2017), CINAHL (EBSCO) (1937–2017), PsycINFO (EBSCO) (1806–2017), Scopus (to 2017), Web of Science Core Collection (SCI-expanded, SSCI, CPCI-S, CPCI-SSH, ESCI) (1900–2017) and the Cochrane library (CENTRAL) (to 2017) (Supplementary File 1). Search strategies were run between 15 and 27 November 2018. Where individual search engines allowed, the strategy was restricted to the English language. Searches were limited to publications up to the end of 2017 to create a clear demarcation for future updates.

Additional references were identified through hand searching key systematic reviews (Supplementary File 1). A search of the randomised evaluations of recruitment interventions available in ORRCA on the 1 October 2020 was also undertaken to identify any studies published before 2018 that assessed retention as a secondary outcome. We did not aim to identify grey literature within this iteration due to the anticipated volume of published literature.

### Inclusion and exclusion criteria

Systematic methods were used to develop a comprehensive database of all retention research relevant to RCTs. Studies describing or evaluating activities, study designs or interventions aimed at addressing retention within health research studies were included. Search strategies were designed to focus on retention within host studies classed as RCTs. However, articles reporting retention within other health research designs such as cohort studies, longitudinal surveys and non-randomised pilot studies that were returned by the searches were included as sources of transferable knowledge and ideas.

For this research, all studies were eligible regardless of their retention rates. Retention at a participant level is defined as continuation in the study and providing data for the required outcomes.

The following topics were outside of the scope of this review:

- Adherence to clinical treatments or research interventions,
- Attendance at appointments outside of research,
- Statistical analysis methods for handling missing data,
- Reporting quality of missing data.

Protocols, commentaries, editorials, letters and news articles were generally excluded as they were unlikely to report primary research results.

We did not assess the quality of included studies, or the journals that their results were presented in; all primary research meeting our inclusion criteria were included in the ORRCA2 database with reference to original publications so that database users can carry out their own quality assessment(s).

### Development of a retention framework for categorising eligible articles

**Figure 1.**
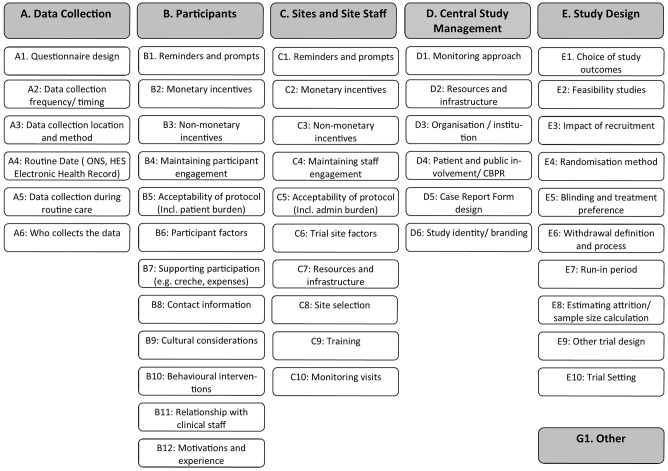
Retention domain framework.

A framework of research themes was developed by grouping previously reported retention themes and strategies^15,17,25^ into domains. Forty-five retention domains were grouped under headings adapted from Palmer et al.:^26^ data collection, strategies aimed at participants, strategies aimed at sites and research staff, central study management, study design and other (Figure 1). The framework was reviewed by co-applicants and members of the MRC Hub for Trials Methodology Research (MRC HTMR) Recruitment and Trial Conduct Working Groups and was piloted on a small number of articles.

### Screening and data extraction

A team of volunteer reviewers was identified through the MRC HTMR, the Health Research Board Trials Methodological Research Network (HRB TMRN) in Ireland and through social media. Reviewers were provided with written guidance and attended teleconference training sessions for abstract screening and full-text review.

Articles were screened by title and abstract by one reviewer. Ten per cent of abstracts assessed by each volunteer reviewer were checked independently as part of quality assurance measures. Full texts were obtained for all potentially relevant articles and were assigned a primary reviewer. Where the primary reviewer was not A.K. (50%), A.K. acted as a secondary reviewer to ensure data extraction consistency. Queries or disagreements were resolved through discussion with a third reviewer (C.G.).

Eligible articles were categorised against as many of the retention domains as relevant and into one of the following types of evidence:

- *Randomised evaluations* of retention strategies,
- *Non-randomised evaluations* of retention strategies (e.g. pre-/post-test),
- *Application* of retention strategies without comparative evaluation,
- *Observations* of factors affecting retention without presenting a formal strategy (e.g. studies exploring patient-reported reasons for early study withdrawal).

Additional information on research methods, host study context and host study population was extracted for eligible articles to facilitate search filters on the website.

Articles initially coded as ‘other’ (G1) were reviewed by two authors (A.K. and N.L.H.) for the possible creation of new themes or re-coding into existing domains.

### Analysis

Descriptive statistics are used to summarise the retention literature across the domains within ORRCA2. The frequencies of retention domains were calculated and presented as a percentage of all articles. Domain frequencies were also calculated for articles categorised within the four different evidence types. Analysis was conducted in SAS 9.4.

## Results

**Figure 2.**
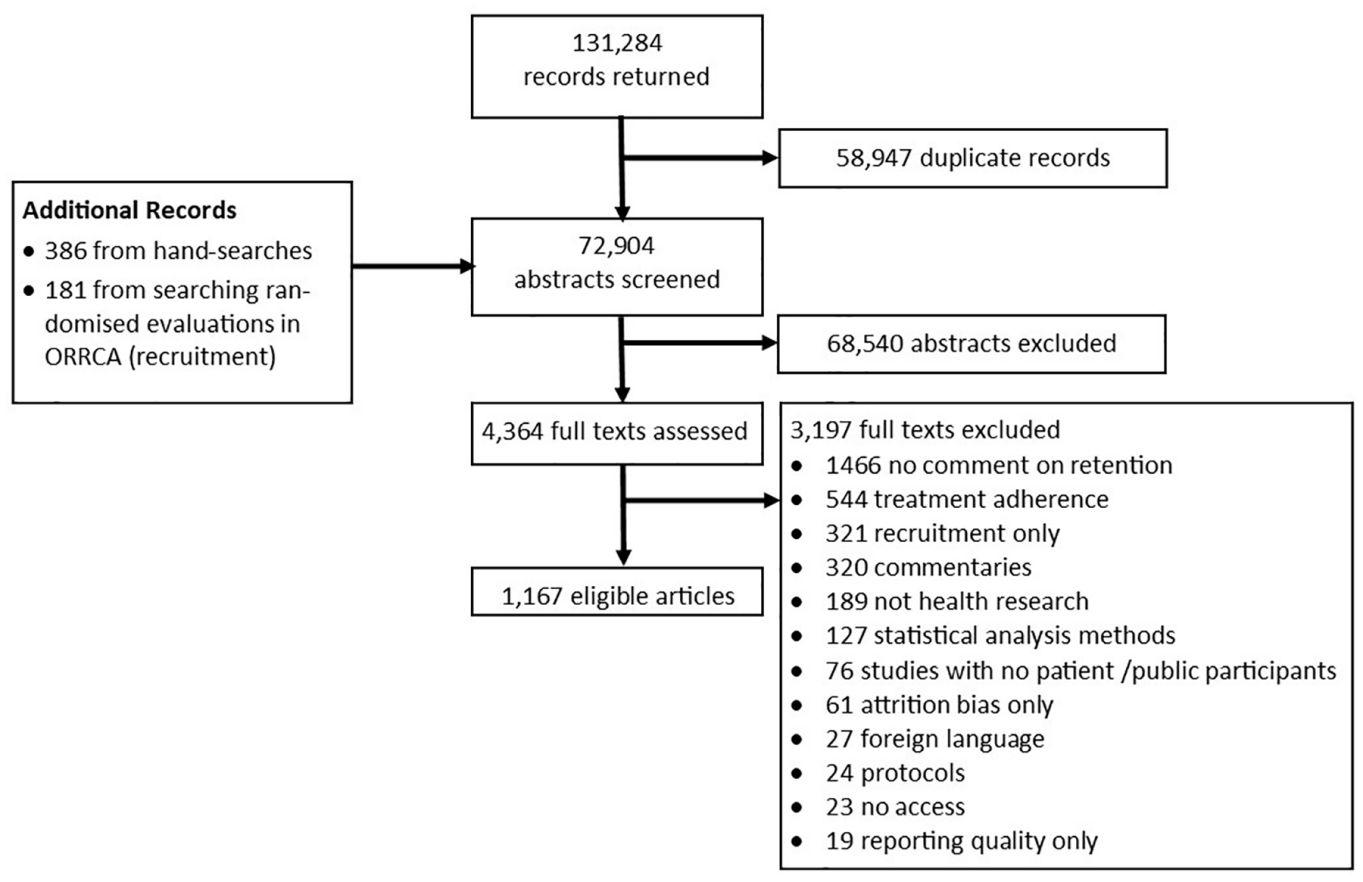
Flow diagram of eligible studies.

Search results returned 131,284 records with an additional 386 unique records identified from hand searches and 181 from a search of recruitment articles available from the ORRCA website (Figure 2). After electronic removal of duplicates, 72,904 records were screened and 4,364 full texts obtained. Of these, 1,167 publications were eligible and uploaded onto a separate searchable database within the ORRCA website.

### Cohort characteristics

Eligible articles were published between 1978 and 2017, although 672 (58%) were published from 2010 onwards (Supplementary Figure 1).

The cohort reported retention within a broad range of clinical studies. However, studies of retention within mental health (292, 25%), cancer (136, 12%) and infection (135, 12%) were most frequently reported (Supplementary Table 1).^7^

Six hundred seventy-one (57%) articles reported retention within parallel RCTs, 44 (4%) within cluster RCTs, 22 (2%) in factorial RCTs and 16 (1%) in crossover RCTs. The cohort included studies conducted across the world, although 501 (43%) were conducted in North America and 303 (26%) in Europe.

Studies most frequently reported recruiting adults aged between 18 and 59 years (626, 54%), but studies including adults over 60 years (341, 29%), children under 16 years (210, 18%) and 16- to 18-year-olds (149, 13%) were still well represented.

Five hundred nineteen studies (44%) were case reports describing retention efforts (Supplementary Table 2). A further 233 (20%) were secondary analysis of the host study, focusing on retention providing increased detail. One hundred fifty (13%) were systematic reviews or reviews and 131 (11%) reported randomised studies of retention interventions that were nested within clinical research.

Nine hundred seventy-nine (84%) of studies assessed the number of participants retained, 317 (27%) demographic differences between lost and retained participants and 233 (20%) questionnaire response rate. Only 44 (4%) assessed costs associated with retention strategies.

Outcome data were collected by questionnaires in 541 (46%) of studies, using clinical measures in 214 (18%) studies and via the provision of blood or tissue samples in 152 (13%) studies (Supplementary Table 1). The location of outcome data collection within the host study was unclear or not reported in 510 studies (44%). In 373 (24%) studies, it was collected from participant’s home, through postal questionnaires, phone calls or by researchers visiting their address. Outcome data were collected through clinic visit in 276 (24%) of studies and via online methods (e.g. emails, websites) in 88 (8%) of studies.

Across the cohort, studies often did not report details of the clinical context and setting within which retention was being assessed. For example, blinding information was not reported in 488/826 (59%) of randomised studies. Information on where follow-up and data collection were undertaken was unknown in 510 (44%) of studies and the type of data collected was also unknown in 371 (32%). In 469 (40%) studies, the recruitment setting was unknown; in 330 (28%) studies, participant ages were unknown and in 266 (23%) studies the country or continent in which the research was undertaken was not reported.

### Domains and evidence type

One hundred sixty-five (14%) of studies were categorised as randomised evaluations, 99 (8%) as non-randomised evaluations of retention strategies, 319 (27%) as an application of retention strategies without evaluation and 584 (50%) were observations of factors affecting retention.

All 45 domains within the framework were used at least once to categorise the eligible articles. Forty-seven papers coded as *G1: Other* were reviewed. Two studies remained in the G1 category and the rest were recoded into existing domains. These reported retention research priorities and publication characteristics associated with different rates of participant retention.

**Figure 3.**
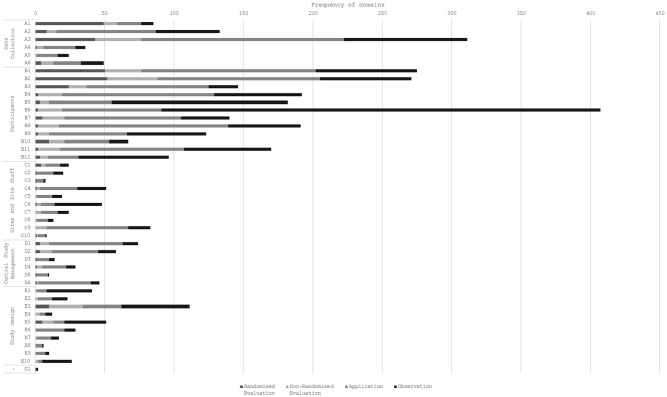
Frequency of retention domains. The full domain names are listed in Figure 1.

Each article could contribute to multiple domains. The number of domains per article ranged from 1 to 18 (median (interquartile range): 2 (1–4)). Figure 3 shows the number of articles within each domain. Across the 1,167 articles, the most frequent domains were *B6: Participant factors* (407, 35%), *A3: Data collection location and method* (311, 27%), *B1: Reminders and Prompts* (275, 24%) and *B3: Monetary incentives* (271, 23%).

**Figure 4.**
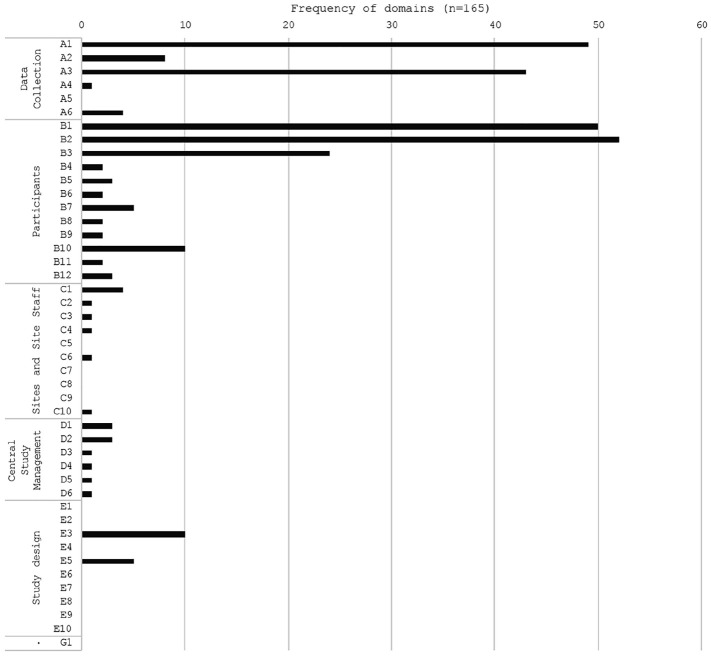
Frequency of retention domains within evidence type ‘randomised evaluations’. The full domain names are listed in Figure 1.

Within the 165 studies categorised as ‘randomised evaluations’, *B2: Incentives* was the most frequently reported domain (52, 32%), followed by *B1: Reminders and prompts* (50, 30%), *A1: Questionnaire Design* (49, 30%) and *A3: Data collection location and method* (43, 26%) (Figure 4). Thirteen retention domains were not reported: *A5: Data collected in routine care*, *C5: Site and site staff acceptability of trial protocol, C7: Resources and infrastructure, C8: Site selection, C9: Site training, E1: Choice of outcomes, E2: Feasibility studies, E4: Randomisation method, E6: Withdrawal definition and processes, E7: Run-in-period, E8: Estimating attrition in sample size calculations, E9: Other trial designs* and *E10: Trial setting.* Of the studies within the randomised evaluation category, 82 (50%) of studies within the randomised evaluations category were assessing retention within RCTs.

## Discussion

Retention research has exponentially increased in the last 10 years. However, identifying relevant methodological research literature is still challenging. The extensive and resource-intensive searching of thousands of abstracts for eligible articles demonstrate the importance of the ORRCA2 resource for trialists and methodologists. Mapping the retention literature highlights the current focus on retention strategies aimed at data collection methods and research participants, the lack of cost–benefit analysis and the need to improve reporting of clinical contexts in publications.

Overall, the cohort of eligible articles covered a broad range of health contexts and demographics. Retention research was most frequently conducted in areas widely acknowledged to be susceptible to attrition such as studies in mental health, studies evaluating behavioural interventions and studies collecting data through questionnaires.^27–29^

Participant reminders and prompts, participant monetary incentives and flexibility around data collection methods were the most frequently reported retention strategies and the focus of most nested randomised studies alongside questionnaire design. This may reflect the growing importance of patient-reported outcomes which are often collected by questionnaire and widely reported to be susceptible to low return rates and missing data.^30,31^ While data collection overseen by research or clinical staff often has higher return rates, the benefits of remote follow-up and alternative data collection methods are becoming increasingly important due to the COVID-19 pandemic.^32^ ORRCA2 will be able to help trialists identify possible solutions to challenges faced for both remote and in-person follow-up through the use of search filters for outcome data type and outcome data collection.

The mapping of the literature highlights several areas for future research. The majority of retention domains that were not reported in the classification ‘randomised evaluations’ were all within the domain headings *C: Sites and site staff* (four domains unreported) and *E: Study design* (eight domains unreported) except *A5: Data collection in routine care*. For domains such as *choice of study outcomes* and *feasibility studies*, randomised evaluations may not be possible, unlike *site selection and site training*. The relationship between clinical staff and participants is often seen as important for retention.^33–35^ In addition, clinical staff need to carefully balance communication about study withdrawal with the effects of attrition to help participants make an informed choice about their ongoing involvement.^36,37^ Consequently, research into clinical staff’s awareness of attrition problems, their role in mitigating it and their training needs should not be overlooked.

In the last 3 years, consensus methods have identified priorities for retention research^17^ and the need to address key uncertainties around retention.^38^ Site training and the use of routinely collected data were in the top three strategies warranting further evaluation but ORRCA2 found no randomised evaluations in corresponding domains C6 and A5. Similarly, several less populated domains mapped directly onto the top 10 retention research priorities identified by the PRioRiTy II study, affirming the limited evidence regarding participant motivations and experience, the use of routine data, administrative burden at research sites and the role of patient and public contributors. In contrast, our domains’ analysis suggests there may already be a wealth of information on different data collection methods. However, the recent update of the Cochrane review of randomised evaluations of retention strategies^39^ has highlighted that even when retention domains are well populated, further evaluation may be still needed if the quality of evidence is not sufficiently high. As such, well-designed evaluations of monetary incentives and electronic reminders are among those being recommended as priorities for future evaluation.

Future research should also consider cost–benefit analysis of retention strategies. While nearly a third of included studies did assess which participants more likely to be retained, very few studies reported the economic cost-effectiveness associated with retention strategies. Analysis of the cost–benefit ratio must be built into future research as this will play an important role in the selection of retention strategies within trials.

Screening and mapping retention literature has also highlighted several challenges and areas for improvement in clinical and methodological research reporting. Information about the host study was often poorly reported, failing to report recruitment methods adequately, data collection location and blinding, all of which may impact retention rates. Retention strategies need to be tailored according to participant demographics and trial design. Improved reporting of the host study design is needed, including descriptions of the interventions and control, participant demographics, health context and trial setting, so results can be contextualised.

Furthermore, retention terminology is ambiguous, poorly defined and inconsistently applied. Articles frequently used terms such as ‘retention’, ‘attrition’ or ‘drop out’ without further information. Where ‘retention’ was defined, this described a range of scenarios, including adherence to treatment protocols and completion of screening processes before randomisation, both of which were outside the scope of this review and reduced the precision of the search strategy. Similarly, many studies only assessed early study discontinuation as a measure of treatment acceptability and therefore did not discuss the perceived causes or potential solutions. Consequently, only 1,167 (27%) of abstracts put forward for full-text review were found to be eligible. In comparison, 57% (4014/7036) of full-text recruitment articles assessed up to the end of 2017 were found to be eligible. Even where articles were within the scope of this review, authors used considerably different criteria for assessing retention, such as outcome data return or availability, attendance at a clinic visit, participant status at a specific point in follow-up or completion of all required research activities. Going forward, ORRCA2 search strategies will be optimised based on the identified literature, and text mining will be utilised in order to efficiently maintain the resource.^40,41^ However, we recommend that in the absence of a universally agreed definitions, authors must clearly describe their criteria and approach to the measurement of retention to allow readers to assess their relevance better.

### Strengths and limitations of research

Extensive searches of multiple databases, along with the hand searching of relevant papers and the ORRCA database, were used to identify retention literature conducted across the world and within all health areas.

Eligible literature was reported in the English language due to lack of funds for translation. However, only 27 studies were excluded on this basis and searches of Scopus, the most comprehensive database,^42^ contained no language restrictions. Therefore, it is unlikely the focus on English language has significantly impacted the results.

The search strategies focused on identifying retention strategies within RCTs but a wider approach was taken during screening, including articles reporting retention to other types of health research. Consequently, the database will not contain a comprehensive account of retention within cohort studies, longitudinal surveys and other non-randomised host study designs.

Studies of interventions to improve visit adherence and data return outside of research may have important transferable knowledge but were not included as this would have made the review unmanageable.

Domain coding was complex and there was some overlap between categories. Reviewers were encouraged to take an inclusive approach and code all relevant domains, but we encourage users of the database to recommend changes or additional coding through the ‘Contact us’ section of the website. As eligible articles were coded with all relevant domains, some of the domains used for studies categorised as ‘randomised evaluations’ may not have been the nested studies’ primary focus. However, as 66% of articles in this category were assigned one domain and 18% were assigned two domains, the impact on the analysis is likely to be minimal.

## Conclusion

ORRCA2 has involved a large-scale literature review of published retention research identifying 1167 eligible papers up to the end of 2017. Mapping the literature highlights a current research focus on retention strategies aimed at participants and data collection methods. Inclusion of cost–benefit analysis in future research and improved reporting of host study context is needed to help with the targeted selection of effective strategies.

## Supplemental Material

sj-docx-1-ctj-10.1177_17407745211053803 – Supplemental material for Developing an online, searchable database to systematically map and organise current literature on retention research (ORRCA2) Click here for additional data file. Supplemental material, sj-docx-1-ctj-10.1177_17407745211053803 for Developing an online, searchable database to systematically map and organise current literature on retention research (ORRCA2) by Anna Kearney, Polly-Anna Ashford, Laura Butlin, Thomas Conway, William J Cragg, Declan Devane, Heidi Gardner, Daisy M Gaunt, Katie Gillies, Nicola L Harman, Andrew Hunter, Athene J Lane, Catherine McWilliams, Louise Murphy, Carrie O’Nions, Edward N Stanhope, Akke Vellinga, Paula R Williamson and Carrol Gamble in Clinical Trials
