# Supplementary material for: Developing an online, searchable database to systematically map and organise current literature on retention research (ORRCA2)
Source: Clin Trials. 2021 Oct 24;19(1):71–80. doi: 10.1177/17407745211053803 (PMC8847754; doi:10.1177/17407745211053803)
Supplement: sj-docx-1-ctj-10.1177_17407745211053803 – Supplemental material for Developing an online, searchable database to systematically map and organise current literature on retention research (ORRCA2) [file sj-pdf-1-ctj-10.1177_17407745211053803.pdf]

Figure S1. Results with $p^*_t = p^*_c$, $\delta_{RD} = -0.1$, one–sided $\alpha = 0.05$, power=0.80

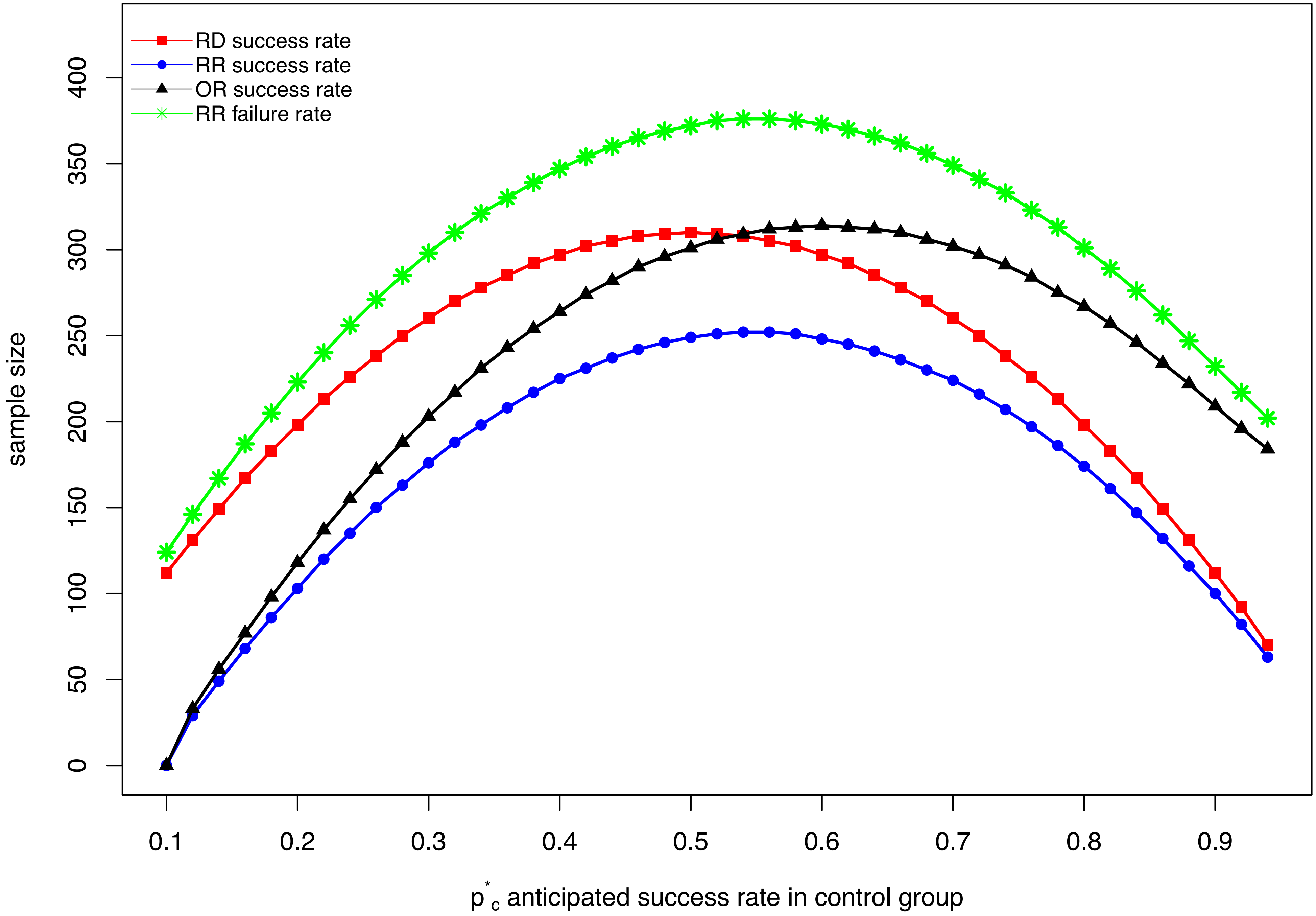

Figure S2. Results with $p^*_t = p^*_c$, $\delta_{RD} = -0.05$, one-sided $\alpha = 0.05$, power=0.80

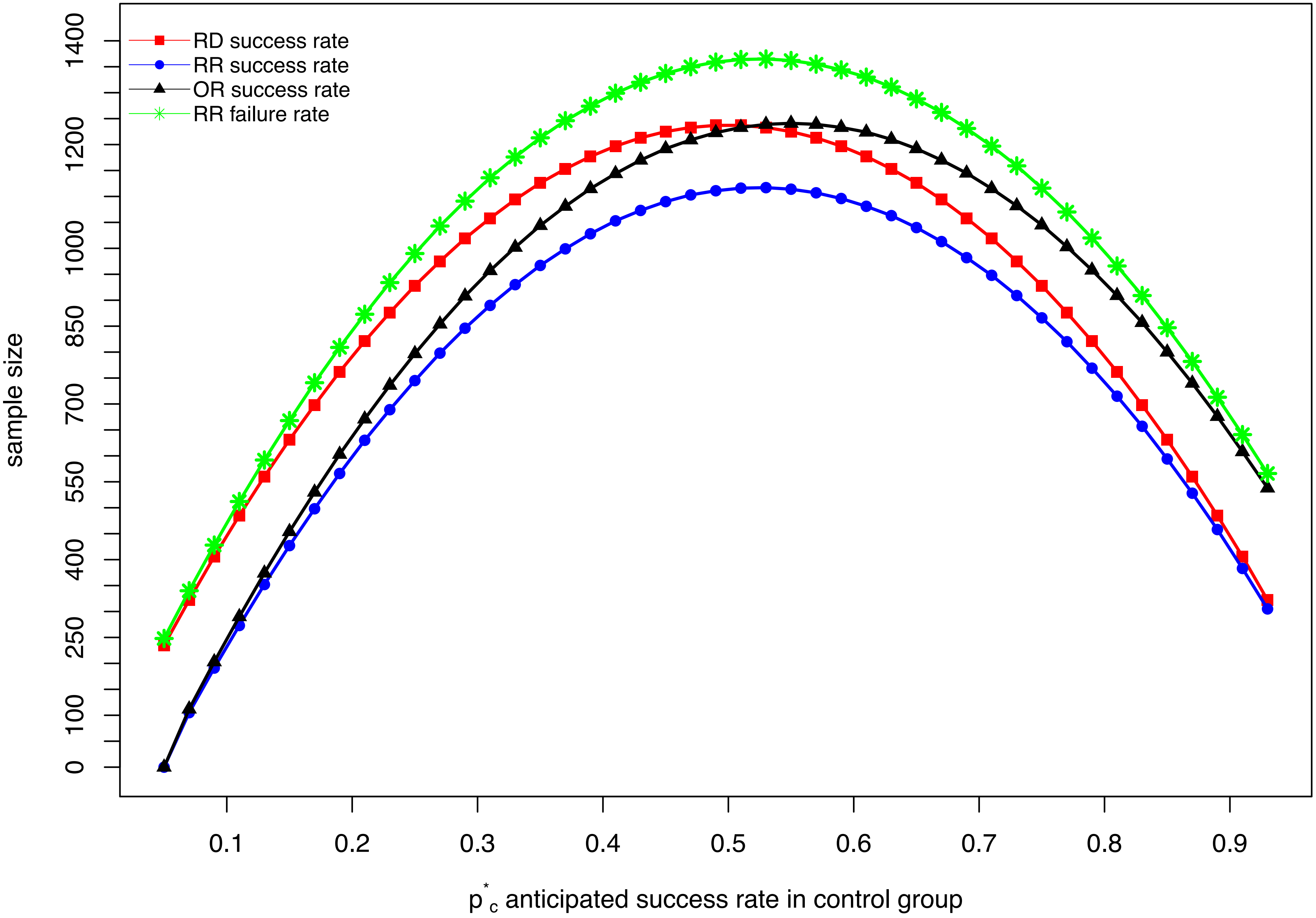

Figure S3. Results with $p^*_t = p^*_c$, $\delta_{RD} = -0.01$, one-sided $\alpha = 0.05$, power=0.80

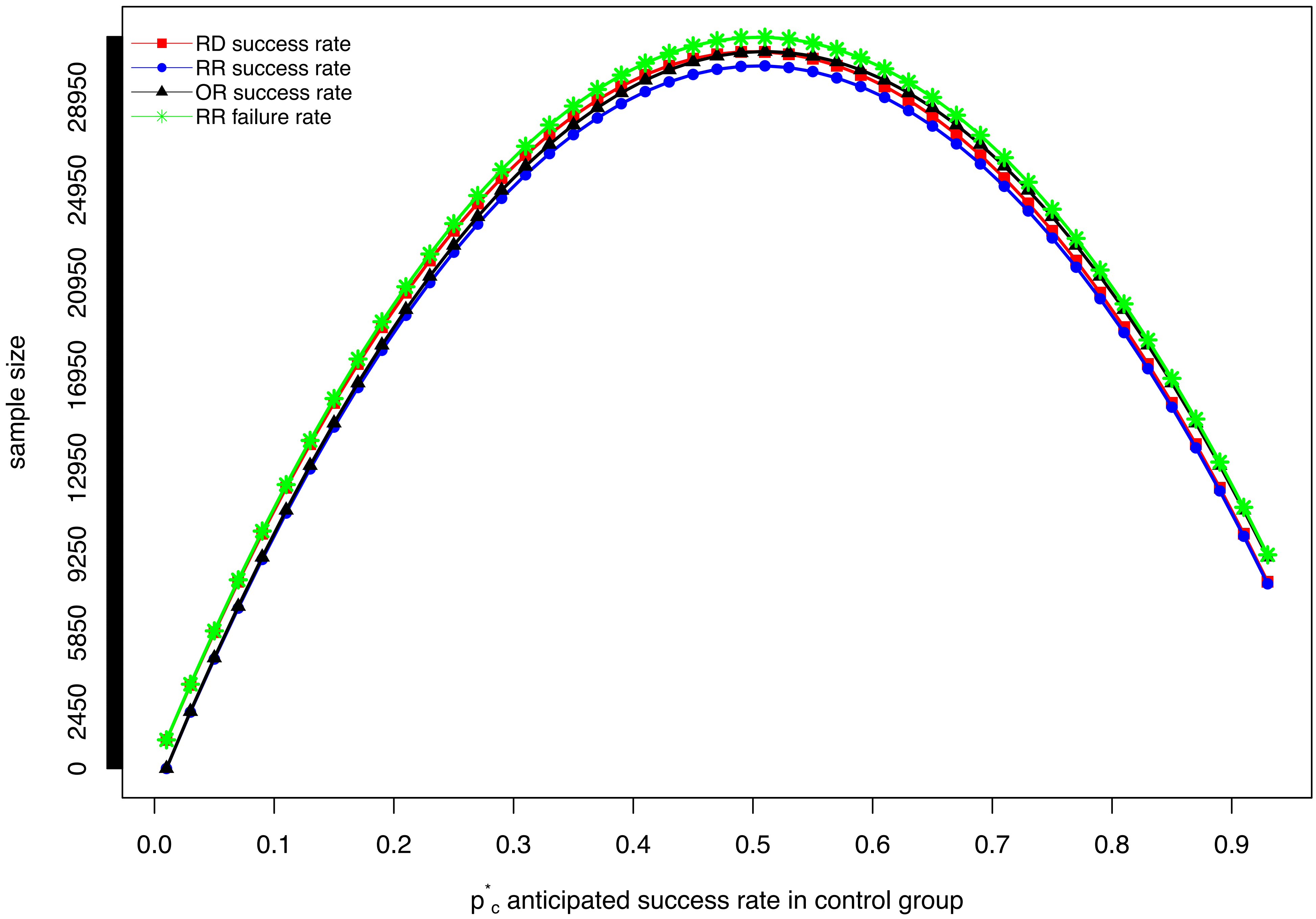

Figure S4. Rejection regions for power when switching using the anticipated control proportion

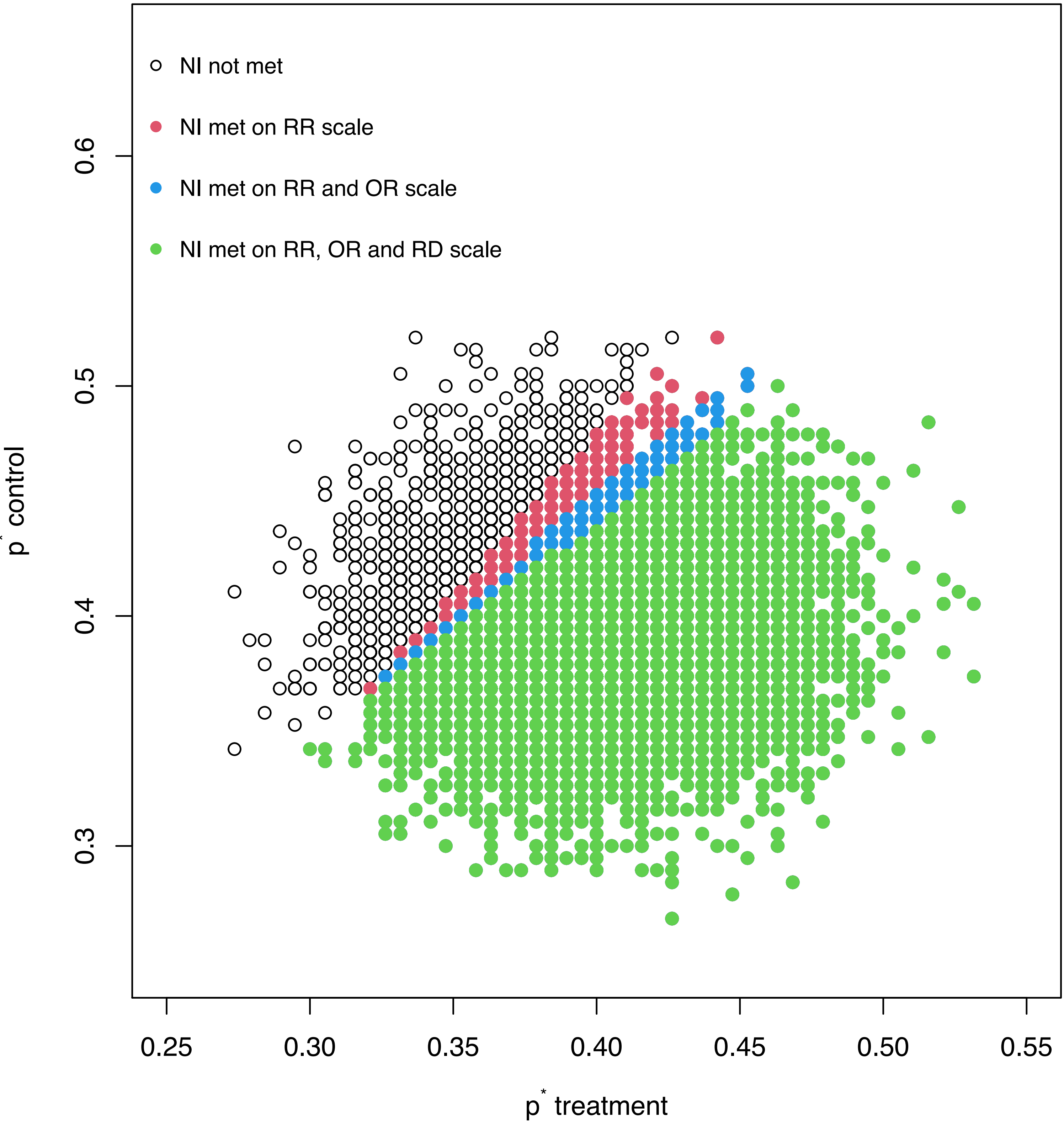

Figure S5. Rejection regions for type I error rate when switching using the anticipated control proportion

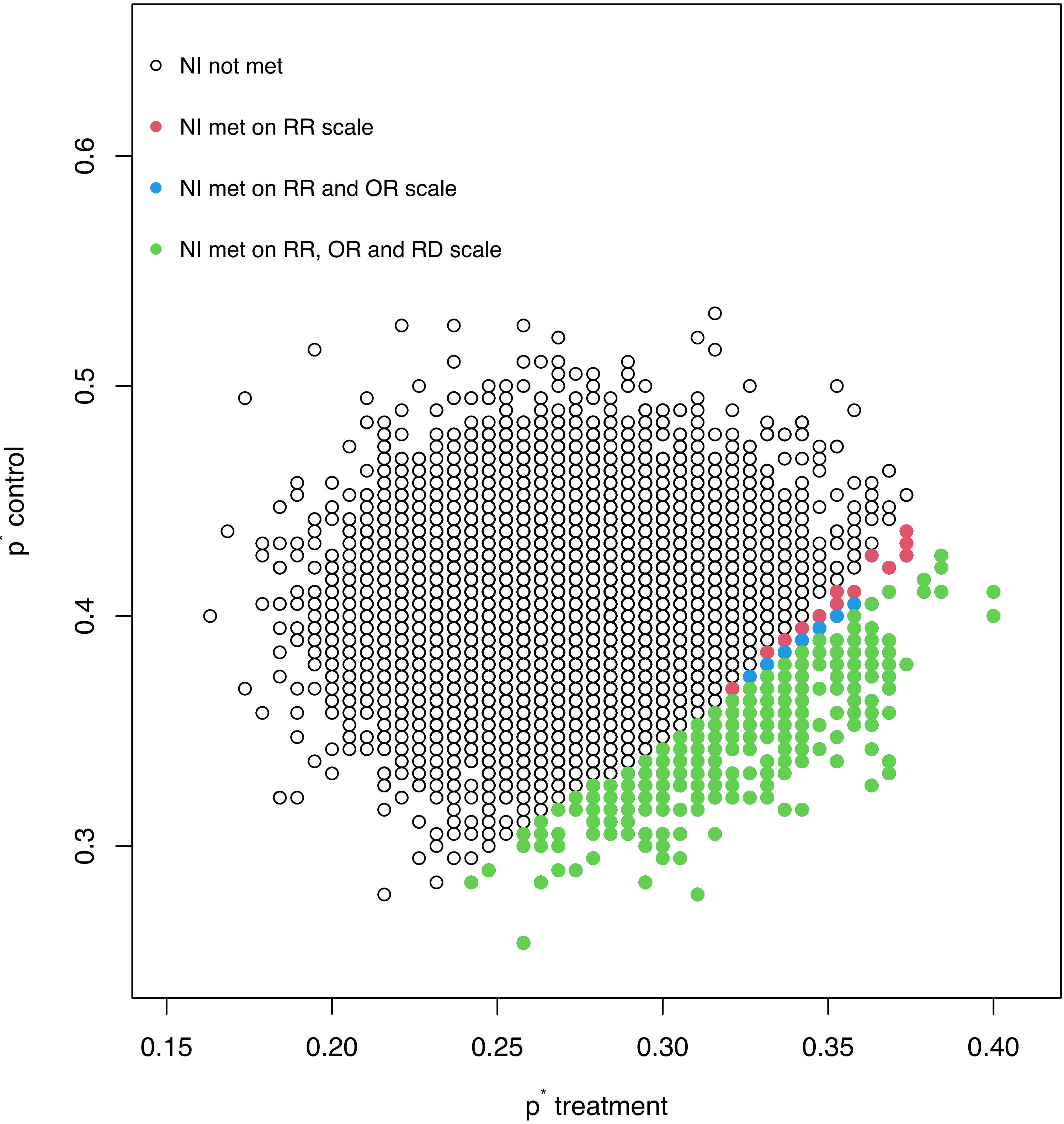

Figure S6. Rejection regions for power when switching using the observed control proportion

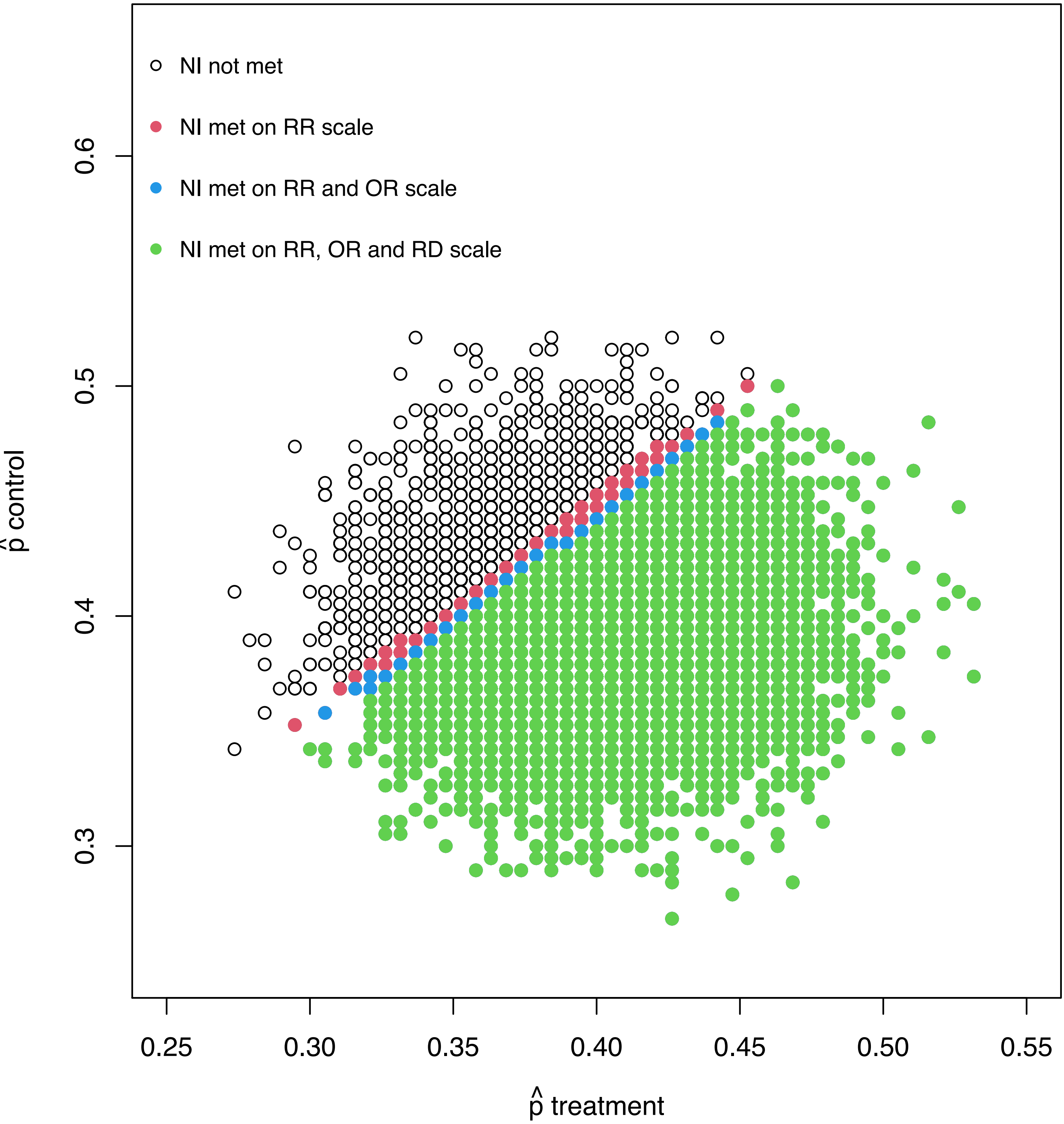

Figure S7. Rejection regions for type I error rate when switching using the observed control proportion

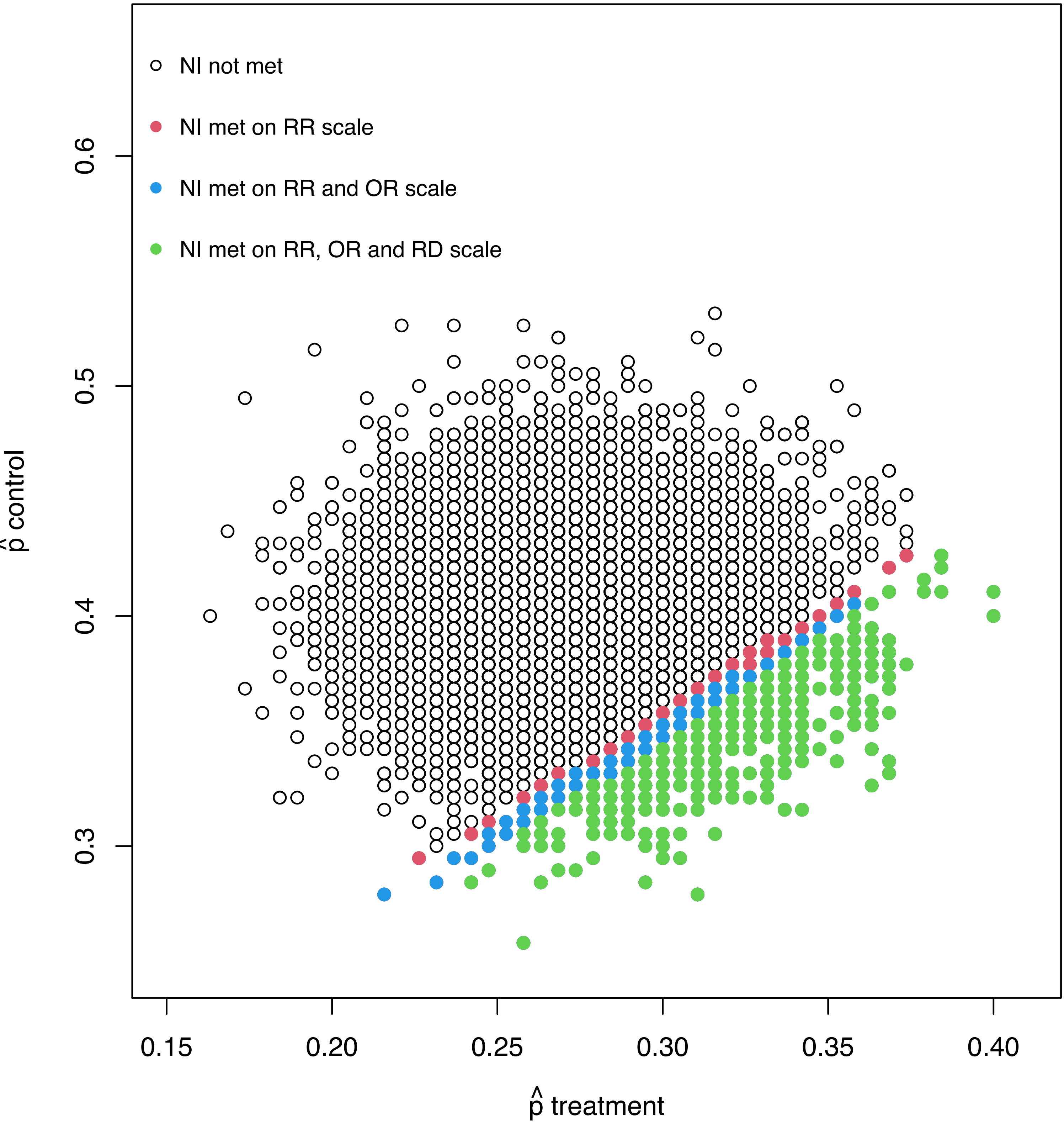

Figure S8. Rejection regions for power when switching using the anticipated control proportion
scenario 1

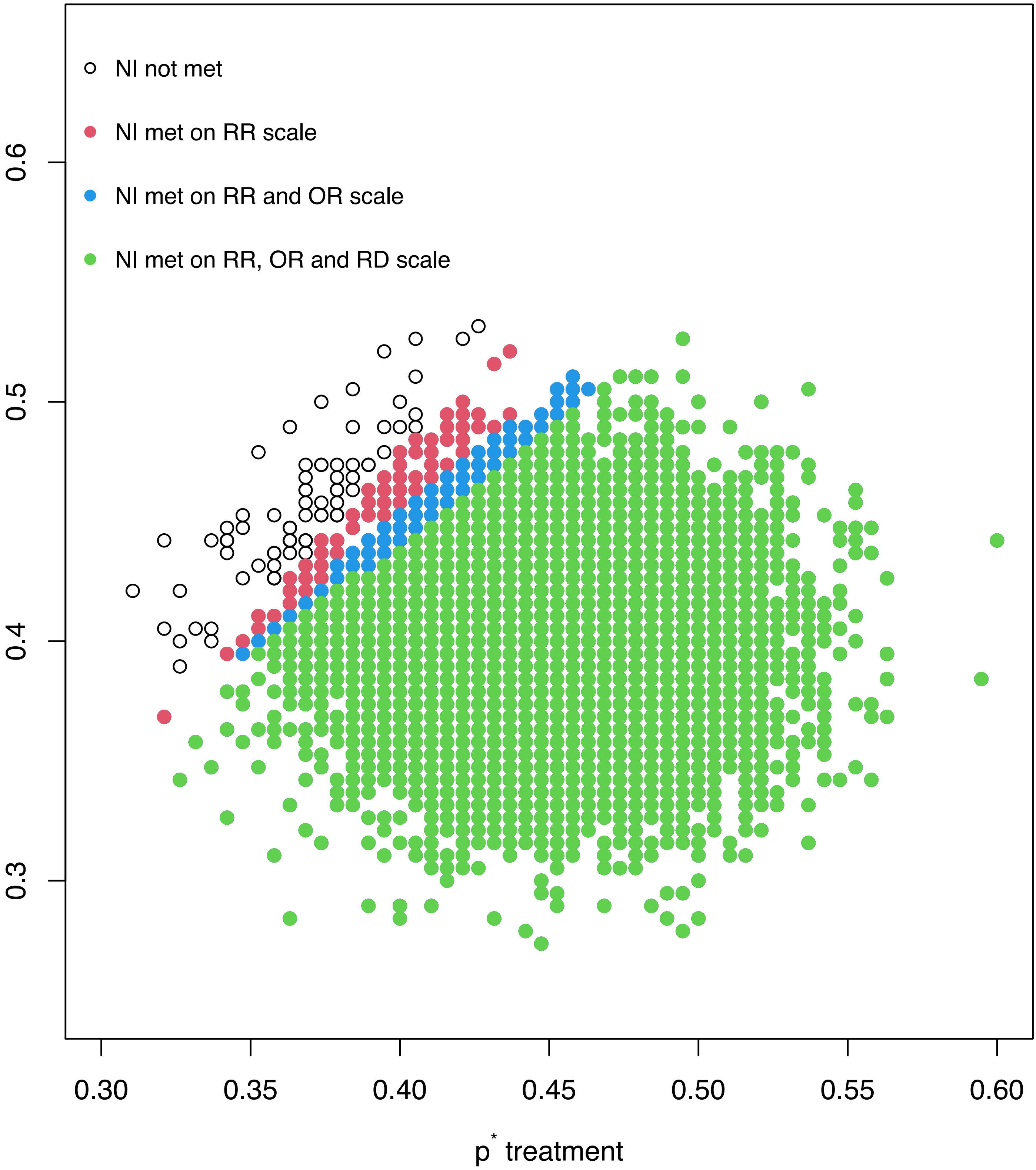

Figure S9. Rejection regions for power when switching using the anticipated control proportion scenario 2

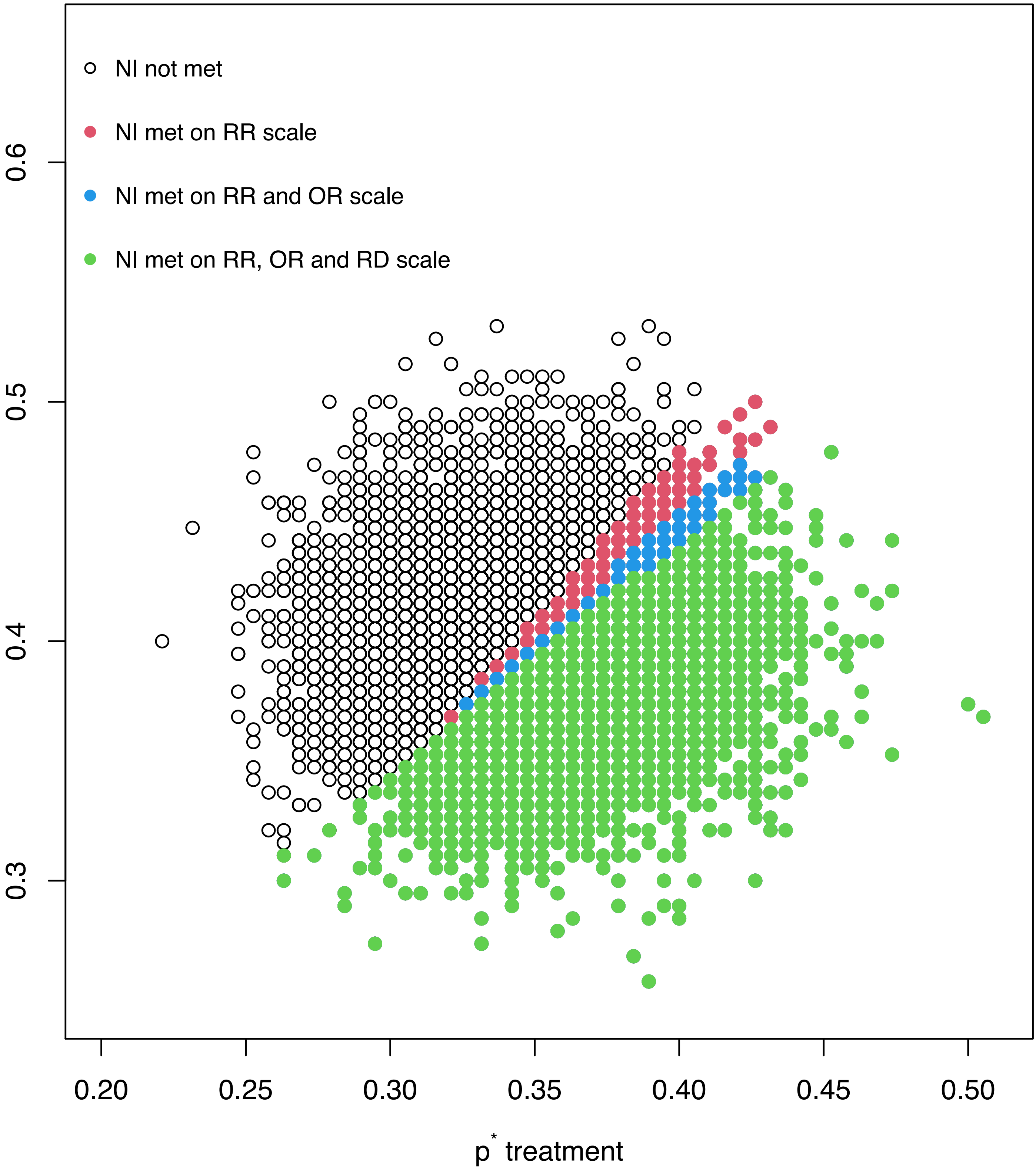

Table S1. Non-inferiority hypotheses and sample size formulas based on Z-tests for four different analysis scales. n represents the sample size required per arm. δ_{RD}, δ_{RR}, δ_{OR} and δ^f_{RR} represent the margin given on the RD, RR, OR scales with success rate and on the RR scale with failure rate, respectively. α denotes the type I error rate, β indicates the type II error rate and $z_{1-\alpha}$ ($z_{1-\beta}$) is the lower α-th (β-th) quantile of the standard normal distribution.

RD	RR (success rate)	OR	RR (failure rate)
$H_0 : p_t - p_c \leq \delta_{RD}$	$H_0 : p_t/p_c \leq \delta_{RR}$	$H_0 : (\frac{p_t}{1-p_t})/(\frac{p_c}{1-p_c}) \leq \delta_{OR}$	$H_0 : (1-p_t)/(1-p_c) \geq \delta^f_{RR}$
$H_a : p_t - p_c > \delta_{RD}$	$H_a : p_t/p_c > \delta_{RR}$	$H_a : (\frac{p_t}{1-p_t})/(\frac{p_c}{1-p_c}) > \delta_{OR}$	$H_a : (1-p_t)/(1-p_c) < \delta^f_{RR}$
$n_{RD} = \frac{2(z_{1-\alpha}+z_{1-\beta})^2 p^*_c(1-p^*_c)}{\delta^2_{RD}}$	$n_{RR} = \frac{2(z_{1-\alpha}+z_{1-\beta})^2 \frac{(1-p^*_c)}{p^*_c}}{(\ln(\delta_{RR}))^2}$	$n_{OR} = \frac{2(z_{1-\alpha}+z_{1-\beta})^2}{(\ln(\delta_{OR}))^2 p^*_c(1-p^*_c)}$	$n^f_{RR} = \frac{2(z_{1-\alpha}+z_{1-\beta})^2 \frac{p^*_c}{(1-p^*_c)}}{(\ln(\delta^f_{RR}))^2}$

Table S2. Given margin on the RD scale (δ_{RD} is negative here), mapping the NI margin to the RR, OR scales using anticipated control rate or observed control rate with success proportions (p_c^* and $\hat{p}_c$) or failure proportions ($(1-p_c^*)$ and $(1-\hat{p}_c)$), respectively. δ_{RR}, δ_{OR} and δ_{RR}^f represent the margin given on the RR,OR scales with success rate and on the RR scale with failure rate, respectively.

Target scale	Using anticipated control rate	Using observed control rate
RR	$\delta_{RR} = (p_c^* + \delta_{RD})/p_c^*$	$\delta_{RR} = (\hat{p}_c + \delta_{RD})/\hat{p}_c$
OR	$\delta_{OR} = 1 + \frac{\delta_{RD}}{p_c^*(1-p_c^*-\delta_{RD})}$	$\delta_{OR} = 1 + \frac{\delta_{RD}}{\hat{p}_c(1-\hat{p}_c-\delta_{RD})}$
RR^f	$\delta_{RR}^f = (1 - p_c^* - \delta_{RD})/(1-p_c^*)$	$\delta_{RR}^f = (1 - \hat{p}_c - \delta_{RD})/(1-\hat{p}_c)$
